# Nutritional and Functional Composition of Barley Varieties From Legambo District, Ethiopia

**DOI:** 10.1155/2024/1367540

**Published:** 2024-10-07

**Authors:** Yalew Yiblet, Worku Misganaw, Endale Adamu

**Affiliations:** ^1^Department of Biology, College of Natural and Computational Sciences, Mekdela Amba University, P.O. Box 32, Tuluawlia, Ethiopia; ^2^Department of Biology, College of Natural and Computational Sciences, Debark University, P.O. Box 90, Debark, Ethiopia; ^3^Department of Biology, College of Natural and Computational Sciences, Debre Tabor University, P.O. Box 272, Debre Tabor, Ethiopia

**Keywords:** antioxidant activity, barely varieties, Legambo District, nutrition

## Abstract

In agriculture, barley holds significant importance as a vital crop with multiple uses. It provides a variety of advantages, including weed suppression, erosion management, nutrient recycling, and improved soil structure. The nutritional and functional composition of barley varieties' samples were analyzed using AOAC methods. The moisture content of the samples ranged from 7.3% to 12.8%, while the ash content varied from 0.5% to 13%. The crude fiber content ranged from 0.5% to 1.5%, and the crude protein content ranged from 0.73% to 3.4%. Furthermore, the crude fat content ranged from 0.11% to 0.8%. The carbohydrate content of the samples were found to be between 69.5% and 82.5%, with an energy value ranging from 338.2 to 382.02 kcal/100 g. In terms of mineral content, the samples exhibited varying levels of calcium (310–670 mg/100 g), iron (34.9–65 mg/100 g), zinc (8.9–16 mg/100 g), and magnesium (Mg) (520–1122 mg/100 g). In addition, the range of the total phenolic content was 1.2 to 3.1 mg/100 g, while the range of the total flavonoid content was 0.41 to 0.55 mg/100 g. Therefore, barley, a selenium-rich food, acts as an antioxidant, protecting cells from free radical damage, reducing inflammation, and reducing the risk of chronic illnesses. The highest content of stearic acid (3.4 g/100 g) followed by myristic acid (2.6 g/100 g) were found in barley varieties. Barley amino acids are essential nutrients for various biological processes, muscle repair, immunological system function, neurotransmitter generation, and detoxification.

## 1. Introduction

Barley (*Hordeum vulgare*) is a cool-season annual cereal grain that plays a crucial role in agriculture, particularly as a cover crop. It provides a variety of advantages, including weed suppression, erosion management, nutrient recycling, and improved soil structure [1].

In Ethiopia, most barley production, specifically about 99.94%, takes place in the country's four primary barley-producing regions, which are predominantly located in the Southern Nations, Nationalities, and Peoples' Regional State [2]. The regions identified as crucial for Ethiopian barley production are crucial for its cultivation and production. At higher elevations, typically between 2000 and 3500 m above sea level, barley exhibits thriving growth. In the elevated Dega regions of Ethiopia, barley assumes the role of a predominant “meher” crop, denoting its significance as a primary season crop. Furthermore, it is widely cultivated as a “belg” crop in numerous localities [3].

The grain is used in many traditional Ethiopian cuisines, including injera, a sourdough flatbread that is a staple food. In addition, barley is used to make stews, soups, and porridges, which are staple foods for the people who live in the highlands of Ethiopia [4].

This crucial information underscores the vital role that barley plays in the food security and sustenance of the local population [5].

Barley's high fiber content can help with weight management and improved digestive health, which is one reason for its usefulness in processed meals. Furthermore, barley has antioxidants such as phenolic compounds that have been connected to a number of health advantages, including a lower risk of chronic illnesses such as cancer and heart disease [6].

The objectives of the investigations were to understand the composition and quality of barley grains, influenced by factors such as rainfall, temperature, soil conditions, fertilization techniques, and agricultural practices [7]. Studies suggest that variations in the chemical makeup of grains belonging to the same variety may be influenced by various factors such as agronomic, climatic, soil, and cultivation practices [8]. Compared to wheat, barley has a lower lipid content and is mainly composed of various fatty acids, including palmitic, oleic, linoleic, and linolenic acid [9]. This cereal crop is rich in the fat-soluble vitamin E and vitamin B complex, which enhances its nutritional profile [10]. Barley amino acids are essential nutrients for various biological processes, muscle repair, immunological system function, neurotransmitter generation, and detoxification.

In the Legambo District, the investigation of the nutritional and phytochemical composition of native barley varieties has not been previously conducted. This study aims to fill this gap in knowledge by analyzing the nutritional content, such as carbohydrates, proteins, fats, vitamins, and minerals, as well as the phytochemical profile of different native barley varieties grown in the region. Therefore, understanding the nutritional and phytochemical composition of these barley varieties can provide valuable information for promoting their consumption for improved health and nutrition outcomes.

## 2. Materials and Methods

### 2.1. Sample Collection and Preparation

The initial phase of this research study entailed the identification and selection of five distinct barley varieties sourced from local farmers in the Legambo District, as shown in Table 1. It is plausible that the researchers established particular criteria for the selection of these barley varieties, taking into account factors such as yield potential, disease resistance, adaptability to local conditions, and their historical significance within the community. Ensuring the integrity of samples during transportation is crucial using appropriate packaging materials that protect against environmental factors such as temperature fluctuations and moisture.

These samples were carefully collected, individually tagged, and transported to the Biology department laboratory at Mekdela Amba University. Once in the laboratory, the samples were manually sorted and cleaned using locally available materials. To ensure uniformity, a grain homogenizer machine was used to homogenize the samples. After the cleaning process, each sample was milled separately using a cyclone sample miller with a 0.5-mm sieve. Subsequently, various parameters of nutrient quality were analyzed for each sample, following the appropriate standard methods. These standardized methods ensure an accurate and reliable assessment of the nutrient quality of barley samples.

### 2.2. Nutritional Analysis

#### 2.2.1. Moisture Contents

A standardized method reported in the literature [11] was used to determine the moisture content of the samples. We carefully weighed 2 g of each sample. After that, the samples were dried for 3 h at 105°C in an air oven. The samples were then kept in a desiccator to avoid absorbing any moisture from the room, and reweighing was repeated until a constant weight was achieved.

(1) $$\begin{array}{c} \text{Moisture }\%=\frac{\left(W_{3}-W_{1}\right)}{W_{2}-W_{1}}\times 100, \end{array}$$

where *W*_1_ is the weight of the empty crucible, *W*_2_ is the weight of the sample and crucible, and *W*_3_ is the weight of the dried sample and crucible.

#### 2.2.2. Crude Fat Contents

The Soxhlet extraction method was used to determine the amount of fat [12]. Before being placed in the Soxhlet extraction tube, each plant sample was weighed on filter paper (Whatman No. 2) and placed in a dry extraction thimble. The crude fat or ether extract was determined using the following formula:

(2) $$\begin{array}{c} \text{Fat }\%=\frac{\text{weight of extracted fat }\left(g\right)}{\text{sample weight}\left(g\right)}\times 100. \end{array}$$

#### 2.2.3. Crude Protein

The micro–Kjeldahl method [13] was used to determine the content of crude proteins in samples of barley variety samples. The percentage of nitrogen was calculated using the following formula:

(3) $$\begin{array}{c} \%\text{ Protein}=\frac{\left(V_{2}-V_{1}\right)\times \text{MHCl }}{W}\times 14\times 6.25\times 100, \end{array}$$

where *V*_2_ is the volume of hydrochloric acid used in the titration (mL) required for the test sample, *V*_1_ is the volume of hydrochloric acid required for the blank test, MHCl is the molarity of hydrochloric acid, W is the weight of the sample, 6.25 is the protein–nitrogen conversion factor, and 14 is the nitrogen atomic mass.

#### 2.2.4. Crude Fiber

The fiber content was determined using AOAC methodologies after combining the sample of barley variety flour with an acetone and ethanol mixture [14].

(4) $$\begin{array}{c} \text{Fibre }\%=\frac{\text{Loss of weight on ignition}}{\text{Weight of the sample}}\times 100. \end{array}$$

#### 2.2.5. Ash Content

The AOAC standard procedure was applied to determine the ash content [15]. In porcelain crucibles, 2 g of each sample was placed, weighed, and burned at 550°C for 30 min in a furnace. After being ashed, the samples were removed and allowed to cool in a desiccator before being weighed.

(5) $$\begin{array}{c} \text{Ash }\%=\frac{\left(W_{3}-W_{1}\right)}{W_{2}}\times 100, \end{array}$$

where *W*_1_ is the weight of the empty crucible, *W*_2_ is the weight of the sample before ashing, and *W*_3_ is the weight of the sample after ashing.

#### 2.2.6. Carbohydrate

A calculation method was employed to determine the carbohydrate content of the sample. The following formula was applied to this calculation: carbohydrate content = 100% (moisture + crude protein + crude fat + ash + crude fiber).

#### 2.2.7. Energy Value

The total energy values in the sample were expressed in kcal/100 g and calculated using the following conversion factors: Lipids (fats) were multiplied by 9 kcal/g, and proteins and carbohydrates were also multiplied by 4 kcal/g.

### 2.3. Mineral Contents

The mineral content of the sample was analyzed according to the AOAC guidelines [16]. Atomic absorption spectrophotometer (AAS) was used. The analysis process of these minerals follows two successive steps.

1. Step 1. Ashing: The process involves washing crucibles with 6N HCl and 10% nitric acid, placing them in an oven and a muffle furnace, weighing a barley flour sample, charring it, ashing it in a muffle furnace, moistening it with deionized water, and finally ashing it. The samples are then cooled, moistened, evaporated, and weighed before being cooled in desiccators.
2. Step 2. Dissolution: In this step, ash was treated with 10 mL of 6N HCl to wet it completely and was carefully taken to dryness on a low temperature hot plate. In addition, 15 mL of 3N HCl was added, and crucible was heated on the hot plate until the solution just boils. After cooling the crucible, a filtration was carried on through a filter paper into a graduated flask. 10 mL of 3N HCI was again added to the crucible containing the filtrate and heated until the solution was just boiled, cooled, and continued to filter into the graduated flask. For calcium determination, 2.5 mL of 10% lanthanum chloride solution was added to the flask, then diluted to 50 mL mark with deionized water. The contents of the flask (sample solution) was cooled and diluted to the mark with deionized water.

Iron, zinc, and calcium hallo cathode lamps were used as a radiation source for each element, and air acetylene gas mixture was used as a source of flame. The flame AAS was optimized for each element, adjusting the hallo cathode lamps for maximum absorbance. Standard solutions were prepared, followed by sample blank solutions, and the system was rinsed to prevent contamination.

Standard solution: Five series of working standard for Fe, Zn, and Ca metal solution were prepared by appropriate dilution of the metal stock solutions (1000 ppm) with deionized water containing 2.4 mL 3 N HCL and 0.5 mL lanthanum chloride in 10 mL volumetric flask used to make the sample and standard matrix similar. Hence, a series of standard solutions of the minerals were prepared from stock solution of 20 ppm for Ca (2, 4, 6, 8, and 10 ppm), 10 ppm for Fe (1, 2, 3, 4, and 5 ppm), and 10 ppm for Zn (0.5, 1, 1.5, 2, and 2.5 ppm). The blank was prepared using the same reagents as the sample, and the instrument was set according to the instruction, and the blank, control, and samples were run. On the other hand, the proportion of magnesium (Mg) was determined using a standard flame emission photometer. The Mg concentration in the sample can be determined by this method, which uses the light that excited Mg atoms' emission.

(6) $$\begin{array}{c} \text{Metal content}\left(\frac{\text{mg}}{100g}\right)=\frac{\left(\text{Cs}-\text{Cb}\right)*V}{10*W}, \end{array}$$

where Cs is the concentration of the sample in ppm, Cb is the concentration of the blank in ppm, *V* is the volume (mL) of the extract, and *W* is the weight (g) of the sample.

### 2.4. Determination of Amino Acid Content

We used the Waters ACCQ Tag chemical package [17] for the analysis of dried and defatted barley samples. The samples underwent a 23-h treatment with continuous boiling 6N hydrochloric acid. The treatment was applied in an oven that was around 110°C in temperature. The hydrolyzate was diluted with a 0.1% formic acid solution to prepare it for analysis. The XTerra MS C18 5 *μ*m, 1 × 100 mm reversed phase HPLC/MS column and the Waters Alliance 2695 and 3100 devices were used to identify amino acids. We used a mobile phase of 90% acetonitrile and 10% deionized water for the extraction and identification of amino acids. The column temperature was kept at 40°C, and the mobile phase flow rate was fixed at 0.5 mL/min. The retention periods and peak regions of the standard amino acid mixture were examined to assess the identity and quantitative analysis of the amino acids.

### 2.5. Free Radical Scavenging Activity

The total antioxidant activity of each sample was determined applying, with minor modifications, the 2, 2-diphenyl-l picrylhydrazyl (DPPH) method reported by Hasan et al. [18]. To keep the DPPH solution for future use, it was carefully prepared, wrapped in aluminum foil, and refrigerated. Five grams of barley flour were weighed for each sample and extracted over a 48-h period using 10 mL of methanol. Furthermore, a 100 mL volumetric flask containing 0.1 mL of extract and 3.9 mL of DPPH solution mixed at a concentration of 6 × 10^−5^ mol/L were mixed and allowed to incubate for 35 min at room temperature. After the incubation period, the absorbance at 517 nm was determined using a UV spectrophotometer. The antioxidant properties were calculated using the following formula.

(7) $$\begin{array}{c} \text{Inhibition }\%=\frac{\left(\text{Blank absorbance}-\text{sample absorbance}\right)}{\text{Blank absorbance}}\times 100. \end{array}$$

### 2.6. Total Phenolic Content

The Folin–Ciocalteu technique reported by Fattahi et al. [19] was used to determine the total phenolic content. In summary, 10 mL of methanol was used to homogenize 5 g of the barley sample into a powder and was subsequently extracted over a period of 48 h. Furthermore, 1 mg/mL of the extract was properly mixed with 4.6 mL of filtered water and 1 mL of Folin–Ciocalteu (1N). After adding 3 mL of sodium carbonate (2%) to the liquid, it was left to stand for 2 h. This was done 3 minutes later. The absorbance at 760 nm was last determined with a UV spectrophotometer.

### 2.7. Total Flavonoid Content

To determine the total flavonoid content of barley samples, a minor modification was made to the method described by Chandra et al. [20]. The sample extract (1 mg/mL) was mixed with 4 mL of distilled water. In addition, 2 mL of 1N NaOH and 0.3 mL of 10% AlCl_3_ were added to the reaction flask. Then, 2.7 mL of distilled water was added and well mixed. Finally, the absorbance at 510 nm was determined.

### 2.8. Statistical Analysis

The results of the nutritional and functional composition of barley varieties were examined using one-way analysis of variance (ANOVA) techniques, presented as the mean ± SE of three measurements, and were determined statistically significant using SPSS version 20.

## 3. Result and Discussion

### 3.1. Proximate Composition of Barley Varieties

One of the most critical determinants of barley's quality and its suitability for various applications, including brewing and food production, is its chemical composition, particularly its moisture content. The chemical composition of the five barley variety samples is outlined in Table 2. Our moisture findings are consistent with the results (8.46%–10.93%) reported by Hussain et al. [21] for barley landraces cultivated in diverse regions of Gilgit-Baltistan. The moisture content in barley plays a significant role in influencing its nutritional value, shelf life, and vulnerability to pests and diseases [22].

Regarding crude fat levels, it was noted that the TB_4_ sample exhibited a significantly lower fat content in comparison to the other samples. However, no substantial differences were observed among the SB_3_, TB_2_, TB_5_, and NB_1_ samples. These findings align with the previous research [23], which indicated that the total lipid content of barley varies between 1.8% and 5.7%. It is suggested that low-fat diets may enhance insulin sensitivity and glycemic control, thereby aiding in the prevention of metabolic syndrome and type 2 diabetes [24].

Indeed, studies have suggested that low levels of crude fat in barley varieties may confer potential protective effects against chronic diseases [23]. In our investigation, we observed that the NB_1_ variety exhibited the highest crude fiber content, quantified at 1.5%. However, it is important to note that this figure is comparatively lower than the findings reported in the previous research [17], which indicated a fiber content of 5.70% in barley grains. The discrepancies in fiber content across studies may be attributed to several factors, including genetic variations among barley varieties, differences in growth conditions, and variations in the analytical methods employed for fiber determination [25].

Barley is also a source of numerous antioxidants, such as vitamin E and selenium. Free radicals, which are unstable molecules, can cause cellular damage and are associated with chronic illnesses such as cancer. Antioxidants play a crucial role in protecting cells from such damage. Furthermore, selenium is instrumental in the processes of DNA production and repair [26].

The results of the crude protein analysis demonstrated significant differences among all samples examined. The TB_5_ variety exhibited the highest crude protein content, recorded at 3.4%. This was followed by the NB_1_ variety at 2.7%, the TB_2_ variety at 1.3%, and the SB_3_ variety at 1% (*p* < 0.05). It is noteworthy to highlight the discrepancy between our findings and those reported by Naibaho et al. [27], which indicated a substantially higher protein content of 12.6%. In general, protein serves as a multifunctional nutrient that is crucial for maintaining overall health and well-being. Sufficient protein intake is essential to support optimal metabolic function, maintain fluid and pH balance, enhance immune system function, and facilitate energy production [28].

The highest ash content was analyzed in TB_5_ (13%), followed by SB_3_ (7%) and NB_1_ (4%), while the lowest value was recorded in TB_4_ (0.5%). This finding was closely related to the results of Hagos Hailu Kassegn [29] in Abyssinian purple wheat. Furthermore, our finding was also consistent with a finding conducted by Wioletta Bie [30] in wheat, triticale, barley, and oat grains. A high ash value in a plant sample typically suggests a rich supply of minerals. This indicates that the plant has absorbed and accumulated a significant amount of essential minerals from the soil during its growth. These minerals play crucial roles in various physiological processes within the plant, including enzyme activation, osmotic regulation, and structural support [31].

Table 2 shows that the results on the carbohydrate content showed a significant difference between the barley varieties collected from the study area *p* (<0.05). The carbohydrate content in TB_4_ provided the highest value (82.5%) followed by NB_1_ (80.7%), TB_2_ (76.2%), SB_3_ (72.7%), and TB_5_ (69.5%). This change might be due to genomic modifications and the environmental conditions under which they grow. Our result is comparable to the previous scenario [32], carried out on barley landraces from the Canary Islands. Furthermore, the energy value is highest in TB_2_ (382.02 kcal/100 g), followed by SB_3_ (387.7 kcal/100 g), TB4 (359.9 kcal/100 g), and TB_5_ (345.6 kcal/100 g). The result is consistent with a previous study [33] found in Adabella barley varieties (383.02 kcal/100 g).

### 3.2. Mineral Contents

**Figure 1:**
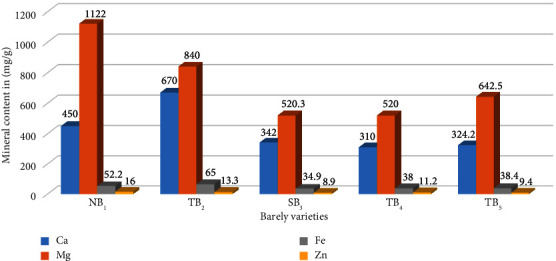
Mineral content of barley varieties in the Legambo District.

Based on the analysis of the mineral compositions of the barley variety, Figure 1 presents the results on the calcium content in various samples. The findings indicate that the calcium content in TB_2_ (670 mg/100 g) and NB_1_ (450 mg/100 g) is significantly higher compared to TB_2_, SB_3_, TB_4_, and TB_5_ (*p* < 0.05). This suggests that the TB_2_ and NB_1_ samples have a markedly elevated calcium content compared to the other samples analyzed. The significant difference observed indicates that the disparity in calcium levels between TB_2_ and NB_1_ compared to TB_2_, SB_3_, TB_4_, and TB_5_ is unlikely to have occurred by chance alone.

In our result, the calcium content was higher compared to other samples collected in different areas from a previous study [34] that recorded the calcium content (0.5 g/kg of DM). Variation may be due to genetic or environmental factors, as well as to the analytical tools used in the analysis. The Mg content in NB_1_ (1122 mg/100 g) was significantly higher than that of TB_5_ and TB_2_ (*p* < 0.05), whereas there were no significant differences between TB_4_ and SB_3_. The study by El-Gohery [35] reported a lower calcium content of 82.92 mg/100 g compared to the results obtained in our analysis. This disparity in the findings suggests that there may be variations in the calcium content of barley samples between the two studies.

According to Figure 1, the zinc (Zn) content of the different varieties of barley showed varying levels of significance. Specifically, the Zn content in NB_1_ was 16 mg/100 g, which was significantly different from TB_2_ (13.3 mg/100 g) and TB_4_ (11.2 mg/100 g) at a significance level of *p* < 0.05. However, no significant differences were observed between SB_3_ (8.9 mg/100 g) and TB_5_ (9.4 mg/100 g). These findings indicate that NB_1_ has a higher zinc content compared to TB_2_ and TB_4_, while SB_3_ and TB_5_ demonstrate similar zinc levels. The significant difference observed between NB_1_, TB_2_, and TB_4_ suggests that there may be variations in the zinc content between these barley varieties in the Legambo District.

Furthermore, the mineral composition of barley varieties can be influenced by ecological conditions and the fertilization system, as highlighted by Sadeghi et al. [36]. Factors such as rainfall, temperature, soil type, and genomic characteristics, as mentioned by Mallikarjuna et al. [37], can also have an impact on the nutrient and physical characteristics of crops, including barley.

### 3.3. The Fatty Acid Composition

Table 3 presents the results of the fatty acid composition, expressed in grams per 100 g (g/100 g), for the various barley varieties. Among the analyzed fatty acids, the highest concentration of stearic acid (3.4 g/100 g) was observed in the NB_1_ variety. The consumption of stearic acid is associated with an increase in fatty acid beta-oxidation, as evidenced by a reduction in circulating long-chain acylcarnitines. This suggests that stearic acid enhances the body's ability to effectively metabolize fats [38].

Subsequently, myristic acid exhibited the second highest content at 2.6 g/100 g, while palmitic acid was measured at 2.5 g/100 g in the SB_3_ variety. These findings indicate that the NB_1_ variety possesses the highest stearic acid content among the barley varieties examined. Stearic acid is a saturated fatty acid commonly found in a variety of foods. Myristic acid, another saturated fatty acid, ranks second in the overall content. Palmitic acid, also a saturated fatty acid, has a slightly lower content but remains noteworthy in the SB_3_ variety. Elevated serum cholesterol levels associated with both myristic and palmitic acids can increase the risk of cardiovascular diseases. However, evidence suggests that myristic acid may exert a more pronounced effect on cholesterol levels than palmitic acid [39].

Our findings are consistent with the study by Tsugumi et al. [38], which found that rice grain barley had a stearic acid content of 0.022 g/100 g, and rolled barley had a myristic acid content of 0.008 g/100 g. These results align with our observation that NB_1_ barley had a higher stearic acid content (3.4 g/100 g) and SB_3_ had a higher myristic acid content (2.6 g/100 g). The fatty acid content of lipoceric acid (0.3 g/100 g) and behenic acid (0.2 g/100 g) in SB_3_, eicosenoic acid (0.2 g/100 g) in NB_1_, and pentadecanoic acid (0.31 g/100 g) in TB_2_ was not found to be significantly different. Limited research exists on the health implications of lipoceric acid, a long-chain saturated fatty acid, but it may share properties with other long-chain saturated fatty acids such as behenic and palmitic acids [40].

These results indicate that when considering these specific fatty acids, the variation between the barley varieties studied was not statistically significant. However, it is important to keep in mind that other fatty acids not mentioned may still exhibit significant differences between the varieties. Our findings are consistent with the results reported by Bouajila et al. [41–44]. Furthermore, in our study, eicosenoic acid was not detected in SB_3_, tetracosenoic acid in TB_2_, pentadecanoic acid in SB_3_, oleic acid in NB_1_, and myristic acid in TB_2_ (Table 3).

### 3.4. Antioxidant Activity of Barley Varieties

Table 4 presents the DPPH radical scavenging activity of the different varieties of barley. The results demonstrate that SB_3_ exhibited significantly higher antioxidant activity compared to samples from other varieties of barley. This finding suggests that SB_3_ has a greater ability to neutralize free radicals and protect against oxidative stress. Antioxidants play a crucial role in maintaining cellular health and reducing the risk of various diseases associated with oxidative damage. The results showed that SB_3_ (49.4%) had the best capacity to eliminate DPPH radicals, followed by TB_4_ (44.33%), TB_2_ (39.4%), NB_1_ (37.3%), and TB_5_ (35.3%). However, there was no significant change (*p* < 0.05) between the varieties NB_1_ and TB_3_. Our findings show a certain degree of similarity to the results reported by the authors in [45], who discovered that approximately 74.1% of Becher wheat varieties showed comparable characteristics. This relative alignment of the findings adds credibility to our study and improves our understanding of Becher wheat varieties. In contrast to the findings reported by the authors in [46], which indicated a DPPH radical scavenging activity of 90.7% in seeds of several varieties of barley, our study observed a comparatively lower DPPH radical scavenging ability. The disparity in the results could be attributed to various factors, including variations in the barley varieties tested, differences in sample preparation, extraction methods, or even variations in the DPPH assay procedure.

### 3.5. Total Components of Phenolics and Flavonoids of Barley Varieties

The total phenolic content of the various varieties of barley is shown in Table 4. There were significant differences (*p* < 0.05) between the results obtained for the parameters tested in each sample. According to current findings, SB_3_ has the highest total flavonoid content (0.6 mg/g), followed by TB_5_ (0.42 mg/g), NB_1_ (0.35 mg/g), TB_4_ (0.3 mg/g), and TB_2_ (0.13 mg/g). The results of the present study were relatively close to those of Simic et al.'s [47] investigation, which found 1.27 mg/g−1.58 mg/g in barely variety grains. According to the study conducted by Dang et al. [48], they recorded a range of 12.94 to 18.65 mg/g for the content of the highland barley varieties. On the contrary, our findings indicate even lower levels of content. This difference in results highlights the variability that can exist in scientific studies. Various factors such as the specific highland barley varieties examined, differences in growing conditions, or variations in analysis methods could contribute to these disparities.

The results of our study indicate that SB_3_ exhibited the highest concentration of total flavonoids, with a recorded value of 0.6 mg/g. On the other hand, TB_2_ had the lowest total flavonoid content, with a measured value of 0.13 mg/g.

The result of our study did not reveal any significant differences between the total flavonoid concentrations of NB_1_ and TB_5_. Both varieties exhibited similar levels of total flavonoids, suggesting that they may have comparable potential health benefits in terms of flavonoid content [49].

In addition to the disparities observed within our own study, it is worth noting that our results were significantly lower compared to the findings of Phuyal et al. [50] in their research on fruit, seed, and bark extracts of *Zanthoxylum armatum*. Similarly, Nowa et al. [51] reported higher values ranging from 10.2 to 29.6 GA mg/kg in conventionally grown black grain barley. These significant variations in the results could be attributed to several factors. First, differences in the species of plants and parts analyzed can lead to variations in the measured GA content. Furthermore, variances in cultivation practices, environmental conditions, and extraction methods used in different studies can contribute to the observed differences [52].

## 4. Conclusions

The findings of the present study indicate that certain barley varieties possess the potential to furnish humans with all essential nutrients. Notably, the varieties TB_5_, TB_4_, and NB_1_ were found to contain significant levels of protein, carbohydrates, fiber, and fats. Furthermore, these barley types have been identified as valuable sources of crucial nutrients, including calcium, zinc, and iron. Given their rich nutritional profiles, these barley varieties are considered suitable for human consumption, provided that appropriate safeguards are implemented to mitigate risks associated with malnutrition. In the course of the antioxidant analysis, the barley varieties TB_2_ and SB_3_ exhibited substantial antioxidant activities, characterized by the presence of phenolic compounds, flavonoids, and free radical scavenging activity. The amino acids derived from barley are critical for various biological processes, including muscle repair, immune system function, neurotransmitter production, and detoxification. The identification of numerous beneficial compounds in these barley varieties underscores their considerable therapeutic potential, suggesting significant implications for the treatment of human ailments.

## Figures and Tables

**Table 1:** The few varieties were analyzed for their approximate composition.

**No.**	**Code for barley varieties**	**Local name barley varieties**
1	NB_1_	Nech barley
2	TB_2_	Tebele barley
3	SB_3_	Senf barley
4	TB_4_	Temj barley
5	TB_5_	Tikur barley

**Table 2:** Proximate composition of five barley varieties.

**Parameters**	**N** **B**_1_	**T** **B**_2_	**S** **B**_3_	**T** **B**_4_	**T** **B**_5_
Moisture	10.6 ± 0.1^c^	12.02 ± 0.01^d^	8.6 ± 0.33^b^	12.8 ± 0.02^e^	7.3 ± 0.03^a^
Fat	0.5 ± 0.02^a^	0.8 ± 0.00^e^	0.11 ± 0.00^b^	0.3 ± 0.00^c^	0.6 ± 0.00^d^
Ash	4.0 ± 0.00^c^	2.0 ± 0.00^b^	7.0 ± 0.00^d^	0.5 ± 0.00^a^	13.0 ± 0.00^e^
Fiber	1.5 ± 0.00^c^	0.5 ± 0.003^a^	0.18 ± 0.02^a^	0.5 ± 0.00^ab^	0.8 ± 0.16^b^
Protein	2.7 ± 0.09^bc^	1.3 ± 0.003^ab^	1.0 ± 0.07^a^	0.73 ± 0.01^a^	3.4 ± 0.7^c^
Carbohydrate	80.7 ± 0.09^d^	76.2 ± 0.03^c^	72.7 ± 0.23^b^	82.5 ± 0.02^d^	69.5 ± 0.8^a^
Energy	338.2 ± 0.05^a^	382.02 ± 0.12^c^	381.7 ± 6.4^a^	359.9 ± 0.07^e^	345.6 ± 0.6^b^

*Note:* Three separate composite sample analyses yielded the mean SD. Significant differences are shown by various superscripts in the column at *p* < 0.05.

**Table 3:** The fatty acid content of various varieties of barley.

**Fatty acid (g/100 g)**	**Barley varieties**				
	**N** **B**_1_	**T** **B**_2_	**S** **B**_3_	**T** **B**_4_	**T** **B**_5_
Lignoceric	1.2	0.8	0.3	0.5	0.5
Behenic	0.6	1.4	0.2	0.6	0.7
Palmitoleic	3.12	1.33	1.4	1.3	2.1
Palmitic	2.4	2.4	2.5	1.6	1.5
Myristic acid	1.7	ND	2.6	1.2	1.4
Stearic acid	3.4	1.3	2.2	ND	2.9
Oleic acid	ND	2.01	1.2	2.3	1.3
Alpha-linolenic acid	3.3	ND	2.1	2.1	1.5
Pentadecanoic acid	2.1	0.31	ND	2.1	0.9
Tetracosenoic acid	1.7	ND	1.9	0.8	1.4
Eicosenoic acid	0.2	1.5	ND	1.4	1.8

ND = not detected.

**Table 4:** The phytochemical content of different varieties of barley.

**Phytochemical constituent**	**Barley variety**				
	**N** **B**_1_	**T** **B**_4_	**T** **B**_2_	**S** **B**_3_	**T** **B**_5_
Total phenolic content (mg/g)	1.4 ± 0.04^e^	2.8 ± 0.12^c^	4.2 ± 0.02^a^	1.1 ± 0.33^d^	3.4 ± 0.13^b^
Total flavonoid content (mg/g)	0.35 ± 0.02^c^	0.3 ± 0.04^d^	0.13 ± 0.05^e^	0.6 ± 0.07^a^	0.42 ± 0.06^ab^
Free radical scavenging activity (%)	37.3 ± 1.4^d^	44.23 ± 2.3^b^	39.4 ± 3.2^c^	49.4 ± 3.2^a^	35.3 ± 1.2^d^

*Note:* Each value represents the mean of three replications ± SD (standard deviation) at *p* < 0.05, means that have different letters from each other are significantly different.

## Data Availability

The data used to support the findings of this study are included within the article.
